# A natural antisense lncRNA controls breast cancer progression by promoting tumor suppressor gene mRNA stability

**DOI:** 10.1371/journal.pgen.1007802

**Published:** 2018-11-29

**Authors:** Mahdieh Jadaliha, Omid Gholamalamdari, Wei Tang, Yang Zhang, Ana Petracovici, Qinyu Hao, Aamira Tariq, Tae Gyoon Kim, Sarah E. Holton, Deepak K. Singh, Xiao Ling Li, Susan M. Freier, Stefan Ambs, Rohit Bhargava, Ashish Lal, Supriya G. Prasanth, Jian Ma, Kannanganattu V. Prasanth

**Affiliations:** 1 Department of Cell and Developmental Biology, Cancer Center at Illinois, University of Illinois at Urbana-Champaign, Urbana, IL, United States of America; 2 Laboratory of human Carcinogenesis, Center for Cancer Research, National Cancer Institute, Bethesda, MD, United States of America; 3 Department of Bioengineering and Beckman Institute of Advanced Science and Technology, Cancer Center at Illinois, University of Illinois at Urbana-Champaign, Urbana, IL, United States of America; 4 Regulatory RNAs and Cancer Section, Genetics Branch, Center for Cancer Research, National Cancer Institute, Bethesda, MD, United States of America; 5 Ionis Pharmaceuticals Inc., Carlsbad, CA, United States of America; 6 School of Computer Science, Carnegie Mellon University, Pittsburgh, PA, United States of America; Universite de Lausanne Faculte de biologie et medecine, SWITZERLAND

## Abstract

The human genome encodes thousands of long noncoding RNA (lncRNA) genes; the function of majority of them is poorly understood. Aberrant expression of a significant number of lncRNAs is observed in various diseases, including cancer. To gain insights into the role of lncRNAs in breast cancer progression, we performed genome-wide transcriptome analyses in an isogenic, triple negative breast cancer (TNBC/basal-like) progression cell lines using a 3D cell culture model. We identified significantly altered expression of 1853 lncRNAs, including ~500 natural antisense transcript (NATs) lncRNAs. A significant number of breast cancer-deregulated NATs displayed co-regulated expression with oncogenic and tumor suppressor protein-coding genes in *cis*. Further studies on one such NAT, *PDCD4-AS1* lncRNA reveal that it positively regulates the expression and activity of the tumor suppressor *PDCD4* in mammary epithelial cells. Both *PDCD4-AS1* and *PDCD4* show reduced expression in TNBC cell lines and in patients, and depletion of *PDCD4-AS1* compromised the cellular levels and activity of PDCD4. Further, tumorigenic properties of *PDCD4-AS1*-depleted TNBC cells were rescued by exogenous expression of *PDCD4*, implying that *PDCD4-AS1* acts upstream of *PDCD4*. Mechanistically, *PDCD4-AS1* stabilizes *PDCD4* RNA by forming RNA duplex and controls the interaction between *PDCD4* RNA and RNA decay promoting factors such as HuR. Our studies demonstrate crucial roles played by NAT lncRNAs in regulating post-transcriptional gene expression of key oncogenic or tumor suppressor genes, thereby contributing to TNBC progression.

## Author summary

Breast cancer is the most common cancer in women worldwide. The molecular mechanisms underlying the disease have been extensively studied, leading to dramatic improvements in diagnostic and prognostic approaches. Despite the overall improvements in survival rate, numerous cases of death by breast cancer are still reported per year, alerting us about the potential gap of knowledge in cancer molecular biology era. The emerging advances in new generation sequencing techniques have revealed that the majority of genome is transcribed into non-protein coding RNAs or ncRNAs, including thousands of long ncRNAs (lncRNAs) of unknown function. Natural antisense RNAs (NATs) constitute a group of lncRNAs that are transcribed in the opposite direction to a sense protein-coding or non-coding gene with partial or complete complementarity. In this manuscript, we investigate the role of NATs in breast cancer progression, focusing on the role of *PDCD4-AS1*, a NAT expressed from the established tumor suppressor *PDCD4* gene locus. We observe that both *PDCD4-AS1* and *PDCD4* display concordant expression in breast cancer cell lines and patients. In mammary epithelial cells, *PDCD4-AS1* promotes the stability of *PDCD4* mRNA. *PDCD4-AS1* by forming RNA duplex with *PDCD4* RNA prevents the interaction between *PDCD4* RNA and RNA decay factors in the nucleus.

## Introduction

While more than 80% of the genome is transcribed to RNA, high throughput gene expression analyses have revealed that only 2% of transcribed RNAs are translated into proteins. Current studies estimate that the human genome harbors several thousands of noncoding RNA (ncRNA) genes [1,2,3,4]. NcRNAs are grouped into different subclasses; from short non-coding transcripts like miRNAs and piRNAs (~20–30 nucleotides [nts] long), to middle range ncRNAs like snRNAs and snoRNAs (~30–200 nts long), and finally the long non-coding RNAs (lncRNAs) (>200 bp in length). So far, the most studied class is microRNAs (miRNAs), which promote gene silencing by inhibiting translation of target genes and/or by destabilizing the mRNAs [5,6]. LncRNAs comprise the least studied, but most complex group of ncRNAs. Unlike miRNAs, lncRNAs are very diverse with respect to their function, localization, abundance and interacting partners [7]. For instance, lncRNAs can form complex 3D secondary structures with the capacity to bind to proteins as well as to nucleic acids (DNA as well as RNA). This dual capacity renders lncRNAs as an ideal regulator in protein-nucleic acid network. The human genome is estimated to contain ~16000 lncRNA genes [https://www.gencodegenes.org]. Based on the genome positioning, lncRNA genes could further be grouped into subclasses, including NATs or natural antisense transcripts (~5501), lincRNAs or long intergenic non-coding RNAs (~7499), sense intronic RNAs (~905), sense overlapping RNAs (~189), and processed transcripts (~544) [https://www.gencodegenes.org].

Breast cancer (BC) is the most common cancer in women, underscoring a need for research and development of more efficient treatment strategies [8]. BC is a heterogeneous disease and comprises several subtypes based on the presence or absence of three hormone receptors; estrogen receptor (ER), progesterone receptor (PR) and human epidermal growth factor 2 (HER2). Based on the expressions of receptors, BC is categorized as Luminal A (ER positive and/or PR positive and HER2 negative), Luminal B (ER positive and/or PR positive and HER2 negative or positive), HER2+ (ER and PR negative, HER2 positive) and triple-negative breast cancer (ER/PR/HER2 negative). The clinical outcome is worst for triple-negative breast cancer (TNBC) patients mainly due to lack of any of the three hormone receptors and, consequently, poor response to hormone-targeted therapies [9,10,11,12]. Therefore, there is an emergent need to investigate the molecular biology of the TNBC subtype to identify efficient prognostic and diagnostic markers.

Current research on BC primarily focuses on the role of protein-coding genes in the disease progression. However, recent studies indicate that a significant number of lncRNAs show aberrant expression in BC patients (For review please see [13]). Abnormal expression of several lncRNAs is associated with chemoresistance in BC cells [14]. However, the underlying molecular mechanism remains to be determined for most cases. Mechanistic studies have indicated that several of the BC-deregulated lncRNAs play crucial roles in disease pathology. For example, HOTAIR is known to negatively regulate the expression of many protein-coding genes by recruiting repressive PRC2 and LSD1 complexes to chromatin. *HOTAIR* is overexpressed in a significant number of BC patients, and is shown to act as a powerful predictor of metastasis [15]. We and others have demonstrated the involvement of *MALAT1* in breast cancer progression and metastasis [16,17]. *MALAT1* is overexpressed in a significant number of BC patients, and its depletion compromises both tumorigenic and metastatic properties of BC cells. In a mouse mammary carcinoma model, genetic loss or systematic depletion of *MALAT1* in MMTV-PyMT resulted in slower tumor growth and reduction in metastasis [16]. In addition to *HOTAIR* and *MALAT1*, both of which promote oncogenesis, lncRNAs such as *GAS5* are shown to act as tumor suppressors [18]. As of now, we understand the molecular action of only a handful of the several thousands of lncRNAs that show aberrant expression in BC patients.

In order to understand the role of lncRNAs during TNBC progression, we performed RNA-seq in an isogenic tumor progressive TNBC cell line series and compared the expression of all of the annotated lncRNAs to a normal-like mammary epithelial cell line. We found that 1853 lncRNAs showed aberrant expression in the metastatic BC cells. Among these lncRNAs, >1/4 (504/1853) of them are found to be natural antisense transcripts (NATs). Interestingly, we observed that several of these NATs are transcribed in opposite orientation to key oncogenic and tumor suppressor protein-coding genes, and the expression of both sense and antisense transcripts is co-regulated in both TNBC cells and BC patient samples. Mechanistic studies of one such NAT, *PDCD4-antisense RNA1* (*PDCD4-AS1*) in BC progression demonstrated that it regulates the expression of its sense protein-coding partner, *PDCD4* (*Programmed Cell Death 4)* in cis. *PDCD4*, initially identified in a screen aimed to determine apoptosis-induced targets [19], is a well-established tumor suppressor gene [20]. We observed that the reduced levels of *PDCD4-AS1* lncRNA in TNBC cells were correlated with reduced expression of *PDCD4* in these cells. Further, we demonstrated that *PDCD4-AS1* acted upstream of *PDCD4* and induced *PDCD4* expression by enhancing the stability of *PDCD4* RNA. Our studies have unearthed novel NAT-mediated post-transcriptional mechanisms controlling the expression of protein coding genes in *cis*.

## Results

### NATs display differential expression during breast cancer progression

Human breast carcinomas are suggested to evolve via sequential genetic modifications from benign hyperplasia of mammary epithelial cells, into atypical ductal hyperplasia, to ducal carcinoma in situ (DCIS) and eventually to fully malignant tumors that possess the potential to metastasize into distant organs [21,22,23]. In order to understand the role of lncRNAs during various stages of breast cancer (BC) progression, we utilized a well-established isogenic mammary epithelial cell line-derived BC progression model system [21,23]. This system consists of multiple cancer cell lines of basal-like or TNBC subtype, all of which were initially derived from the spontaneously immortalized, non-tumorigenic mammary epithelial cell line, MCF10A [24]. The model system comprises of 4 isogenic cell lines, categorized as M1-M4 [21,23]. M1 represents the normal, non-tumorigenic, immortalized MCF10A cells. Transfection of MCF10A with activated T24-HRAS and selection by xenografting generated the M2 (MCF10AT1k.cl2) cell line, which is highly proliferative and gives rise to premalignant lesions with the potential for neoplastic progression. M3 (MCF10Ca1h) and M4 (MCF10CA1a.cl1) were derived from occasional carcinomas arising from xenografts of M2 cells. M3 gives predominantly well-differentiated low-grade carcinomas on xenografting, while M4 gives rise to relatively undifferentiated carcinomas and colonizes to the lung upon injection of these cells into the tail vein [22,25,26,27,28,29]. These lines represent progression through various stages of breast tumorigenesis and recapitulate key steps that mimic the progression of breast cancer *in vivo* [25]. In addition, the common genetic background of these cells enables us to rule out the genetic variation behind the deregulated gene expression. We hypothesized that functional characterization of lncRNAs, especially those displaying differential expression among these cell lines, would help us to determine their roles in TNBC development.

**Fig 1:**
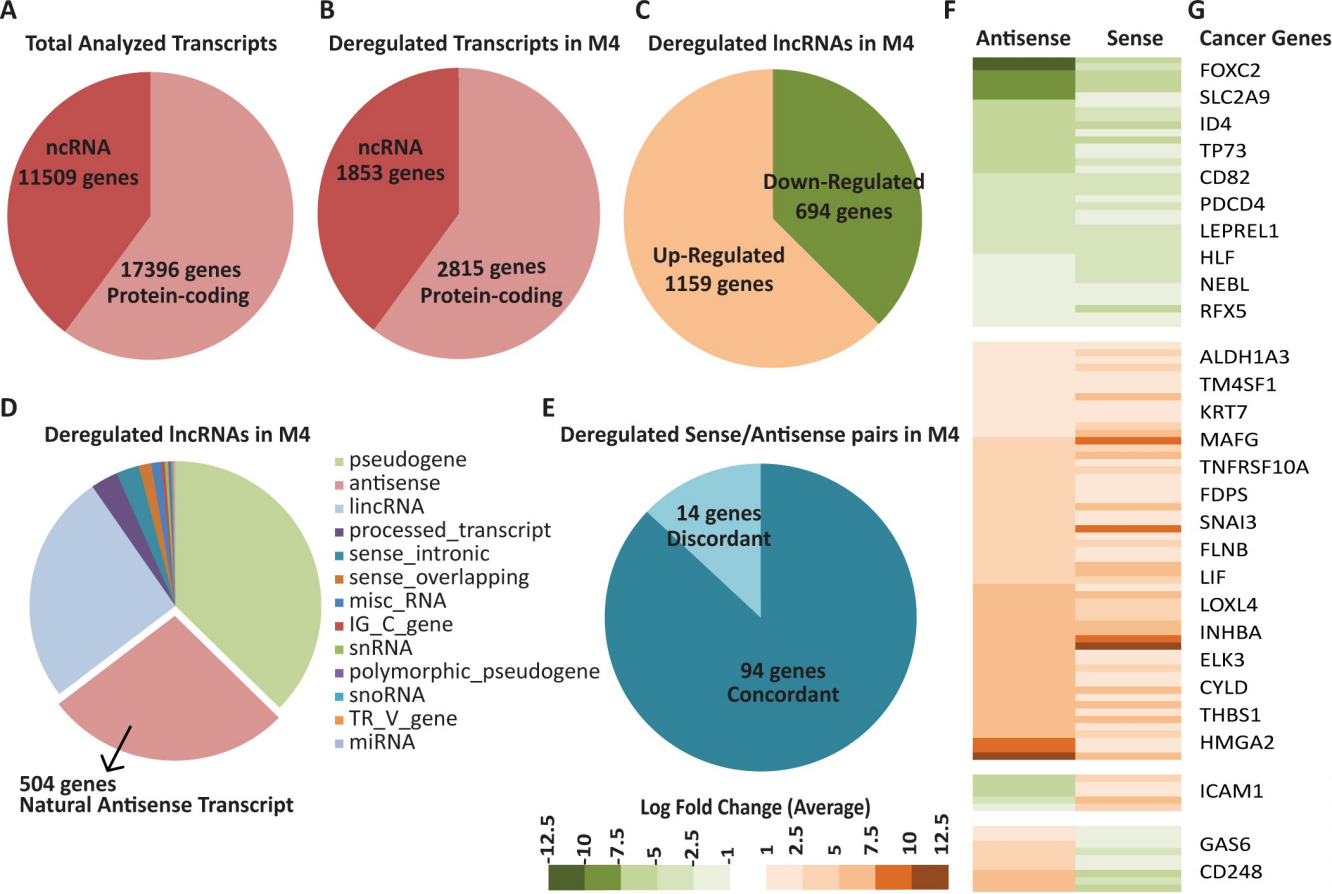
Long noncoding RNAs, including NATs show differential expression during breast cancer progression. A) Total number of genes analyzed in M1 and M4 cells. B) Number of deregulated ncRNA and protein-coding RNA genes in M4 cells compared to M1 cells. C) Number of deregulated lncRNA genes in M4 compared to M1 cells. D) Different classes of aberrantly expressed lncRNA genes in M4 compared to M1 cells. E) Number of sense/antisense pairs that show concordant or discordant pattern of expression in M4 compared to M1 cells. F) Heatmap representing deregulated sense/antisense pairs in M4 compared to M1. G) Representative cancer-associated protein coding genes in the NAT/protein-coding gene pairs from the heatmap (Fig 1F). Please see S6 Table for the details of NAT/protein-coding gene pairs.

We cultured M1-M4 cells as three-dimensional (3D) acinar or organoid-like structures in Matrigel for 7–10 days, as 3D acini structurally and morphologically resemble *in vivo* acini of breast glands and lobules [28,30]. We performed poly A+ selected paired-end deep RNA-seq (~160–250 million reads/sample) in two biological replicates and analyzed the expression of 28905 genes in M1, M2, M3 and M4 cells (17396 protein coding and 11509 lncRNAs) (GENCODE Release v19 [GRCh37]) (Fig 1A). We identified transcripts, which were more than 2-fold deregulated in both biological repeats. Since we were primarily interested in lncRNAs that show abnormal expression during BC progression and metastasis, we initially compared gene expression between M1 and M4 cells (S1 and S2 Tables). Expression of 4668 genes (2815 protein coding and 1853 lncRNAs) were altered >2-fold change in their expression between M1 and M4 cells in both biological repeats (Fig 1B). 1159 out of the 1853 deregulated lncRNA genes showed >2-fold upregulation in M4 cells (Fig 1C, S2 Table). On the other hand, 694 lncRNA genes displayed reduced expression in M4 compared to M1 cells. Further, we noticed that natural antisense transcripts (NATs) comprised one of the largest types of lncRNAs (504 out of 1853), along with lincRNAs and pseudogenes, which showed deregulation in M4 cells (Fig 1D). Our data supports observations from a recent study, reporting deregulated expression a significant number of NATs in breast cancer samples [31].

NAT lncRNAs are typically enriched in the nucleus [1,32,33], and recent studies indicate that several of the NATs function in *cis* by regulating the expression of their sense partner protein-coding genes (for review please see [31,33,34]). To gain insights into the potential NAT-mediated *cis*-gene regulation in BC cells, we examined the status of co-regulated expression of 504 NATs and their protein-coding partner in M1 and M4 cells. We observed that 108 out of 504 deregulated NATs and their sense protein coding genes showed >2-fold change in expression (S3 and S4 Tables). Among them, 94 (~87%) NAT: mRNA pairs showed concordant pattern of deregulation (i.e., both sense/antisense pairs are up- or are down-regulated concordantly) and 14 (~13%) pairs exhibited discordant pattern of expression (Fig 1E and 1F). To assess if these NATs potentially regulate the expression of protein-coding genes that play crucial roles in BC progression, we determined the percentage of the sense protein coding genes in the sense: NAT pair that play well-established roles in cancer progression. We compiled data sets from multiple sources to identify potential cancer-associated genes, that are involved in vital cellular processes such as cell cycle and Epithelial-to-Mesenchymal transition (EMT) (https://www.qiagen.com), (http://www.bushmanlab.org/links/genelists), [17,35] (S5 Table). By such analysis, we identified 29 deregulated NAT: mRNA pairs in which the protein coding genes have established roles in cancer progression (Fig 1F and 1G, S6 Table). Furthermore, comparison of expression data of these NATs with ‘clinical survival in invasive breast carcinoma patient dataset’ (TCGA dataset, containing 105 normal samples and 814 breast tumors) revealed that the expression of 3 of these NATs was well correlated with survival outcomes in BC patients (S7 Table) [36]. Thus, BC deregulated NAT: sense protein-coding genes could potentially play vital roles in BC progression and survival.

### *PDCD4-AS1* is downregulated during breast cancer progression, and its expression positively correlates with *PDCD4* in BC cells and patients

**Fig 2:**
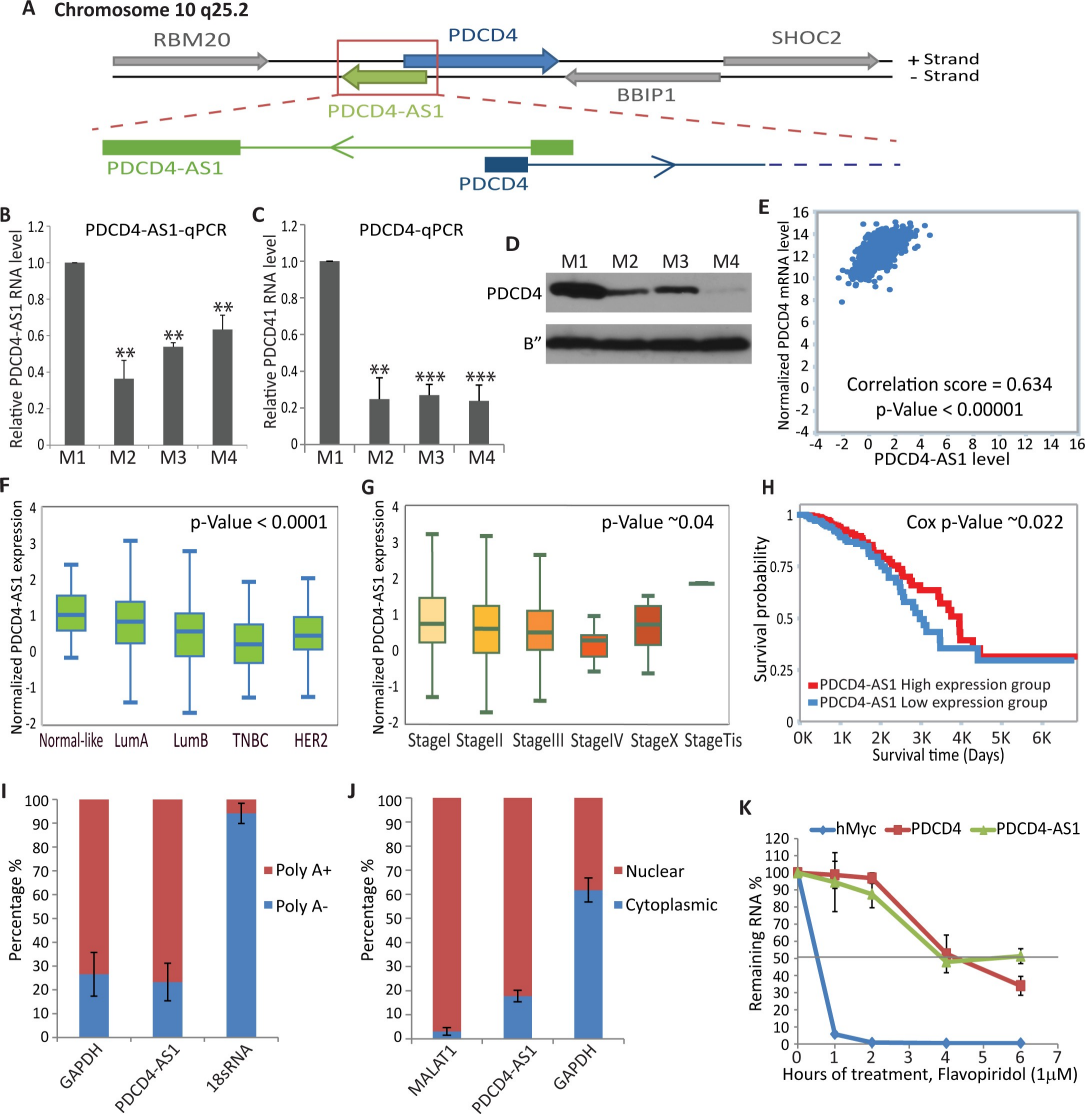
*PDCD4-AS1* is a stable nuclear-enriched lncRNA that shows concordant pattern of expression with its coding partner *PDCD4*. A) Schematic representation of *PDCD4/PDCD4-AS1* gene locus. B) *PDCD4-AS1* RNA level measured by RT-qPCR in M1- M4 TNBC cells. C) *PDCD4* mRNA level measured by RT-qPCR in M1- M4 TNBC cells. D) Immunoblot analysis shows the relative levels of PDCD4 protein in M1- M4 cells. E) Correlation analysis between *PDCD4* and *PDCD4-AS1* RNA in TCGA breast cancer dataset, analyzed by TANRIC platform. F) *PDCD4-AS1* RNA level in different subclasses of breast cancer patients, analyzed by TANRIC platform. G) *PDCD4-AS1* RNA level in different stages of breast cancer patients analyzed by TANRIC platform. H) Kaplan–Meier analysis to depict the survival rate in TCGA breast cancer patients with high and low levels of *PDCD4-AS1*, analyzed by TANRIC platform. I-J) RT-qPCR analyses in poly A^+^ and poly A^-^ (I) and nuclear and cytoplasmic fractionated RNA (J) from M1 cells. K) RT-qPCR to quantify the stability of *PDCD4-AS1* and *PDCD4* mRNA using RNA from M1 cells treated with Flavopiridol (1M) for indicated time points (0, 1,2,4 and 6 hrs). Error bars in (B, C, J & K) represent mean ± SEM of N≥3 independent experiments (biological replicates). *P<0.05, ** P< 0.01 and ***P<0.001 using Student’s t test.

To gain insights into the role of NATs in BC progression, we focused our attention on one NAT lncRNA, *PDCD4-AS1* for the following reasons. *PDCD4-AS1* is a NAT lncRNA, transcribed from the complementary strand of *Programmed Cell Death 4 (PDCD4)* gene (Fig 2A). *PDCD4* is a known tumor suppressor gene that negatively regulates cell proliferation, neoplastic transformation and tumor invasion [37]. RNA-seq, RT-qPCR and immunoblot analyses demonstrated reduced levels of *PDCD4-AS1*, *PDCD4* mRNA and protein in M2, M3 & M4 cells compared to M1 (Fig 2B–2D & S1A and S1B Fig). Furthermore, *PDCD4* and *PDCD4-AS1* RNAs showed significant positive correlation with each other in breast cancer patient RNA data set (Fig 2E). Further, gene expression data from breast invasive carcinoma patients (TCGA data set) [36] revealed that *PDCD4-AS1* showed lowest levels in basal-like or TNBC patients compared to Luminal A, Luminal B and HER2 subtypes (Fig 2F). Highest levels of *PDCD4-AS1* were observed in stage Tis (stage 0, pre-cancer) breast samples compared to samples from the more aggressive stages of BC (Fig 2G). Finally, the elevated levels of *PDCD4-AS1* were correlated with better survival rate in a cohort of BC patients (Fig 2H). Similar to *PDCD4-AS1*, TNBC patient samples showed lowest levels of *PDCD4* mRNA, and higher *PDCD4* mRNA levels correlated with better survival in BC patients, further supporting its role as a potential tumor suppressor (S1C and S1E Fig). Our results indicate that the levels of *PDCD4-AS1* and *PDCD4* mRNA are co-regulated in BC cell lines and in BC patients. Low expression of *PDCD4-AS1* in BC patient samples as well as better survival of patients with higher levels of *PDCD4-AS1* implies that *PDCD4-AS1*, similar to its sense partner *PDCD4*, might function as a tumor suppressor.

RNA-seq and RT-qPCR analyses in M1 cells determined *PDCD4-AS1* as a multi-exonic (two exons), ~778 nts long polyadenylated transcript (S1F Fig & S2I Fig). CPAT algorithm (Coding Potential Assessing Tool) identified *PDCD4-AS1* as a noncoding RNA, as its coding potential score was relatively low and comparable to other well-established lncRNAs such as *MALAT1* (S1G Fig). Further, cellular fractionation followed by RT-qPCR assays indicated that *PDCD4-AS1* lncRNA was enriched in the nuclear fraction in mammary epithelial cells (Fig 2J). Finally, we determined the turnover rate of *PDCD4-AS1* in M1 cells. RNA stability assay indicated that *PDCD4-AS1* is a relatively stable transcript, and it displayed similar stability to its protein-coding partner *PDCD4* mRNA (t_1/2_ of ~4hrs; Fig 2K). Our results identify *PDCD4-AS1* as a stable, poly A^+^ lncRNA that is enriched in the nucleus.

*PDCD4* was initially identified as a tumor suppressor gene that was upregulated during serum starvation or cellular quiescence [19]. To test whether *PDCD4-AS1* is also induced under conditions that activate *PDCD4*, we determined the expression of *PDCD4* and *PDCD4-AS1* in asynchronous and quiescent (serum-starved) M1 cells (S2A Fig and S2B Fig). RT-qPCR and immunoblot data revealed elevated levels of both *PDCD4* (mRNA and protein) and *PDCD4-AS1* RNA in quiescent cells (S2C Fig & S2D Fig). Our results indicate that *PDCD4-AS1* shows co-regulated expression with its protein-coding partner *PDCD4*.

### PDCD4-AS1 negatively regulates cell migration of mammary epithelial cells

**Fig 3:**
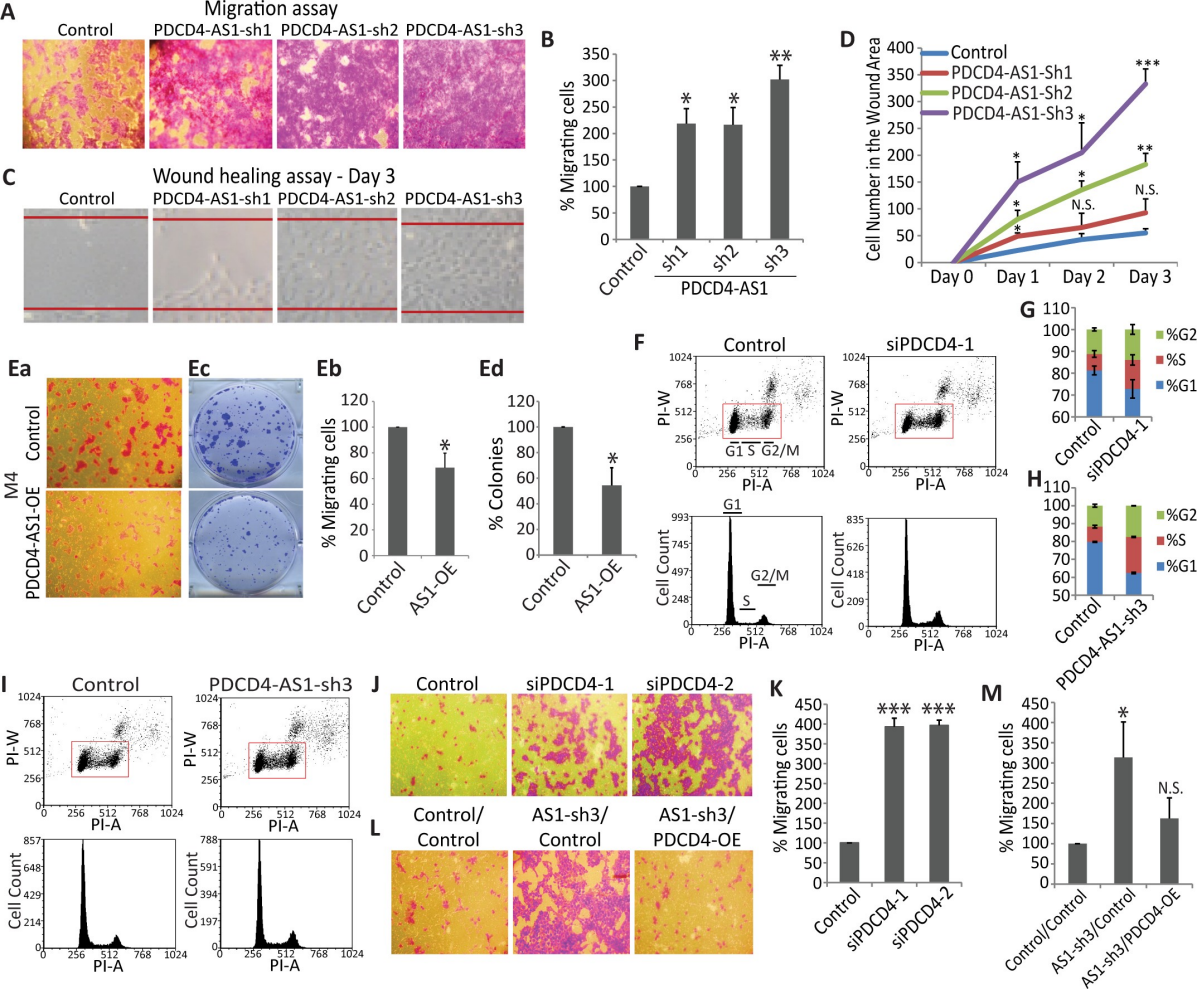
*PDCD4-AS1* negatively regulates cell proliferation and migration. A-B) Transwell migration assay in control and *PDCD4-AS1*-depleted M1 cells. C-D) Wound healing assay in control and *PDCD4-AS1* depleted M1 cells. Images were taken at Day 0, 1, 2 and 3 after wound creation; the magnified image of Day 3 in control and *PDCD4-AS1* depleted cells is shown in the figure C. Ea,b) Transwell migration assay in M4 cells that are overexpressing *PDCD4-AS1*. Ec,d) Long-term anchorage-dependent plastic colony formation assay in M4 cells upon *PDCD4-AS1* overexpression. F-G) Cell cycle flow cytometry in control and *PDCD4-*depleted M1 cells. H-I) Cell cycle flow cytometry in control and *PDCD4-AS1*-depleted M1 cells. J-K) Transwell migration assay in control and *PDCD4*-depleted M1 cells. L-M) Transwell migration assay in control and *PDCD4*-overexpressing M1 cells that are depleted of *PDCD4-AS1* (AS-sh3). Error bars in (B, D, Eb, Ed, G, H, K & M) represent mean ± SEM of N≥3 independent experiments (biological replicates). *P<0.05, ** P< 0.01 and ***P<0.001 using Student’s t test. NS depicts not significant results.

Since a lower level of *PDCD4-AS1* RNA was associated with poor survival in breast cancer patients, and since it showed positive correlated expression with the tumor suppressor gene *PDCD4* both in breast cancer cells and in patients, we evaluated whether *PDCD4-AS1* contributes to cancer-associated phenotypes. We stably depleted *PDCD4-AS1* transcripts by using three independent shRNAs targeting the sequences of *PDCD4-AS1* (exon 2) that were not overlapping with *PDCD4* mRNA (S3A Fig & S3B Fig) in non-tumorigenic mammary epithelial (M1) cells. RT-qPCR revealed that *PDCD4-AS1* shRNA successfully depleted both nuclear and cytoplasmic pool of *PDCD4-AS1* (S3C Fig). Next, we analyzed the migration potential of control and *PDCD4-AS1*-depleted cells. M1 cells depleted of *PDCD4-AS1* showed enhanced migration as observed by both transwell migration and wound healing assays (Fig 3A–3D). Next, we overexpressed the full length *PDCD4-AS1* in highly tumorigenic and metastatic M4 cells (M4 cells contain lower levels of endogenous *PDCD4-AS1*) and determined the effect on cell migration and long-term cell proliferation. We observed that *PDCD4-AS1*-overexpressing M4 cells showed significant reduction in their ability to migrate (Fig 3Ea-b) and displayed reduced proliferation (Fig 3Ec-d). It is known that tumor suppressor PDCD4 inhibits cell proliferation [38]. Flow cytometric analyses revealed increased population of S and G2/M in *PDCD4*-depleted M1 cells (Fig 3F and 3G). Similarly, *PDCD4-AS1*-depleted M1 cells also showed increased population of S and G2/M cells (Fig 3H & 3I). Collectively, these results indicate that both *PDCD4* and *PDCD4-AS1* negatively regulate cell proliferation in human mammary cells.

We observed that depletion of *PDCD4-AS1* increased cell cycle progression, and migratory properties of M1 cells. Depletion of *PDCD4* is also known to promote tumorigenic properties of human cells (For review please see [37]). Similar to what we observed upon depletion of *PDCD4-AS1*, *PDCD4*-depleted M1 cells also showed enhanced cell cycle progression and cell migration (Fig 3F and 3G & Fig 3J and 3K). Based on this, we hypothesize that *PDCD4-AS1* negatively regulates tumorigenic properties of cells via modulating the expression of *PDCD4*. To determine whether *PDCD4-AS1* acts upstream of *PDCD4*, we exogenously expressed of *PDCD4* in *PDCD4-AS1*-depleted M1 cells and tested the effect on cell migration phenotype (S4A Fig). Trans-well migration assays revealed that M1 cells transiently overexpressing *PDCD4* alone did not show any significant change in their ability to migrate *in vitro* (S4A Fig), while *PDCD4-AS1*-depleted control cells displayed increased migration (Fig 3A and 3B & L [left and middle panels]). In contrast, overexpression of *PDCD4* in cells that were stably depleted of *PDCD4-AS1* rescued the enhanced migration, as these cells showed comparable levels of migration to control cells (Fig 3L and 3M; compare left and right panels in 3L). Based on these results, we hypothesize that *PDCD4-AS1* negatively regulates cellular migration via modulating *PDCD4* expression/activity.

### *PDCD4-AS1* promotes the stability of *PDCD4* mRNA

**Fig 4:**
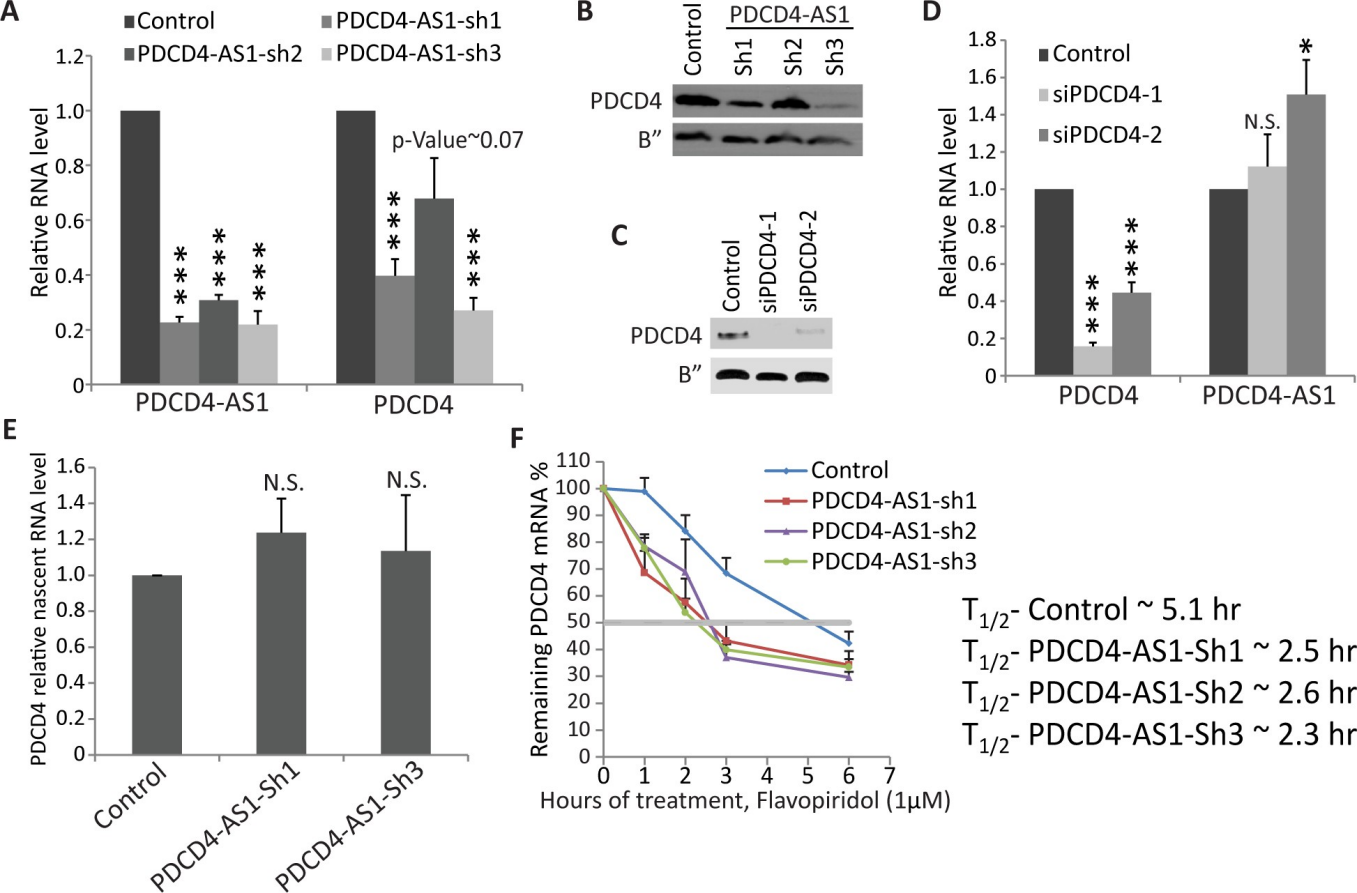
*PDCD4-AS1* promotes the stability of *PDCD4* mRNA. A) *PDCD4* and *PDCD4-AS1* RNA level measured by RT-qPCR in control and *PDCD4-AS1*-depleted M1 cells. B) PDCD4 protein level in control and *PDCD4-AS1*-depleted M1 cells. C) *PDCD4* and *PDCD4-AS1* RNA level measured by qPCR in control and *PDCD4*-depleted M1 cells. D) PDCD4 protein level in control and *PDCD4*-depleted M1 cells. E) Nascent RNA assay in control and *PDCD4-AS1*-depleted M1 cells. F) RT-qPCR to quantify *PDCD4* mRNA stability assay using RNA from control and *PDCD4-AS1*-depleted M1 cells treated with Flavopiridol (1M) for indicated time points (0, 1,2,3 and 6 hrs). Error bars in (A, D, E & F) represent mean ± SEM of N≥3 independent experiments (biological replicates). *P<0.05, ** P< 0.01 and ***P<0.001 using Student’s t test. N.S. represents not significant result.

To determine whether *PDCD4-AS1* negatively regulates cell proliferation and cell migration by regulating the expression of *PDCD4* in *cis*, we examined the level of *PDCD4* mRNA and protein in M1 cells stably depleted of *PDCD4-AS1* using shRNAs. We observed that *PDCD4-AS1*-depleted cells showed consistent reduction in the levels of *PDCD4* mRNA and protein (Fig 4A & Fig 4B). In addition, cells depleted of *PDCD4-AS1* using modified antisense DNA oligonucleotides (GAPMER ASOs) against *PDCD4-AS1* also showed reduction in the levels of *PDCD4* mRNA (S3D Fig). Also, cells treated with *PDCD4-AS1* specific ASOs displayed cell cycle defects that were similar to *PDCD4-AS1* shRNA-treated cells (S3E Fig). In addition, cell fractionation followed by RT-qPCR in control and *PDCD4-AS1*-depleted cells showed significant reduction in the levels of *PDCD4* in the nuclear pool, supporting the argument that *PDCD4-AS1* primarily functions in the nucleus (S3F Fig). Cells depleted of *PDCD4* using two independent *PDCD4* specific siRNAs did not show similar decrease in the levels of *PDCD4-AS1* transcript (Fig 4C & 4D). In case of *PDCD4-AS1*-mediated regulation of *PDCD4*, we tested whether depletion of *PDCD4-AS1* also alters the expression of other genes located in close genomic proximity. RT-qPCR analyses revealed that the expression of several other genes (*BBIP1*, *SHOC2 and RBM20* [Fig 2A] that are located in genomic regions close to *PDCD4-AS1/PDCD4* locus remained unaltered upon *PDCD4-AS1* or *PDCD4* depletion (S4B Fig & S4C Fig). These results imply that *PDCD4-AS1* positively and specifically regulates the expression of its sense transcript.

NATs could regulate the expression of their sense partner genes either by influencing transcription or by modulating post-transcriptional processing of sense transcripts (for review please see [33]). To determine whether *PDCD4-AS1* regulates the transcription of *PDCD4* gene, we quantified the levels of nascent *PDCD4* pre-mRNA in control and *PDCD4-AS1*-depleted cells by nascent RNA capture followed by RT-qPCR analysis. *PDCD4-AS1*-depleted M1 cells did not show any significant change in the total levels of nascent *PDCD4* pre-mRNA, indicating that *PDCD4* transcription remained unaffected in cells lacking *PDCD4-AS1* (Fig 4E). Next, to test whether *PDCD4-AS1* influenced post-transcriptional processing of *PDCD4* mRNA, we performed RNA stability assay. We treated control and *PDCD4-AS1*-depleted cells with an RNA polymerase II transcription inhibitor Falvopiridol (1μM), collected samples at several time points post drug treatment, and performed RT-qPCR analyses to determine the relative levels of *PDCD4* mRNA. Control cells displayed a half-life of ~5 hrs for *PDCD4* mRNA (Fig 4F). However, cells depleted of PDCD4-AS1 showed ~50% reduction in the stability of *PDCD4* mRNA (half-life ~2.5 hrs) (Fig 4F). These results indicate that *PDCD4-AS1* positively regulates the stability of *PDCD4* mRNA.

### *PDCD4-AS1* promotes *PDCD4* mRNA stability by modulating the association of RNA-binding proteins to *PDCD4* mRNA

**Fig 5:**
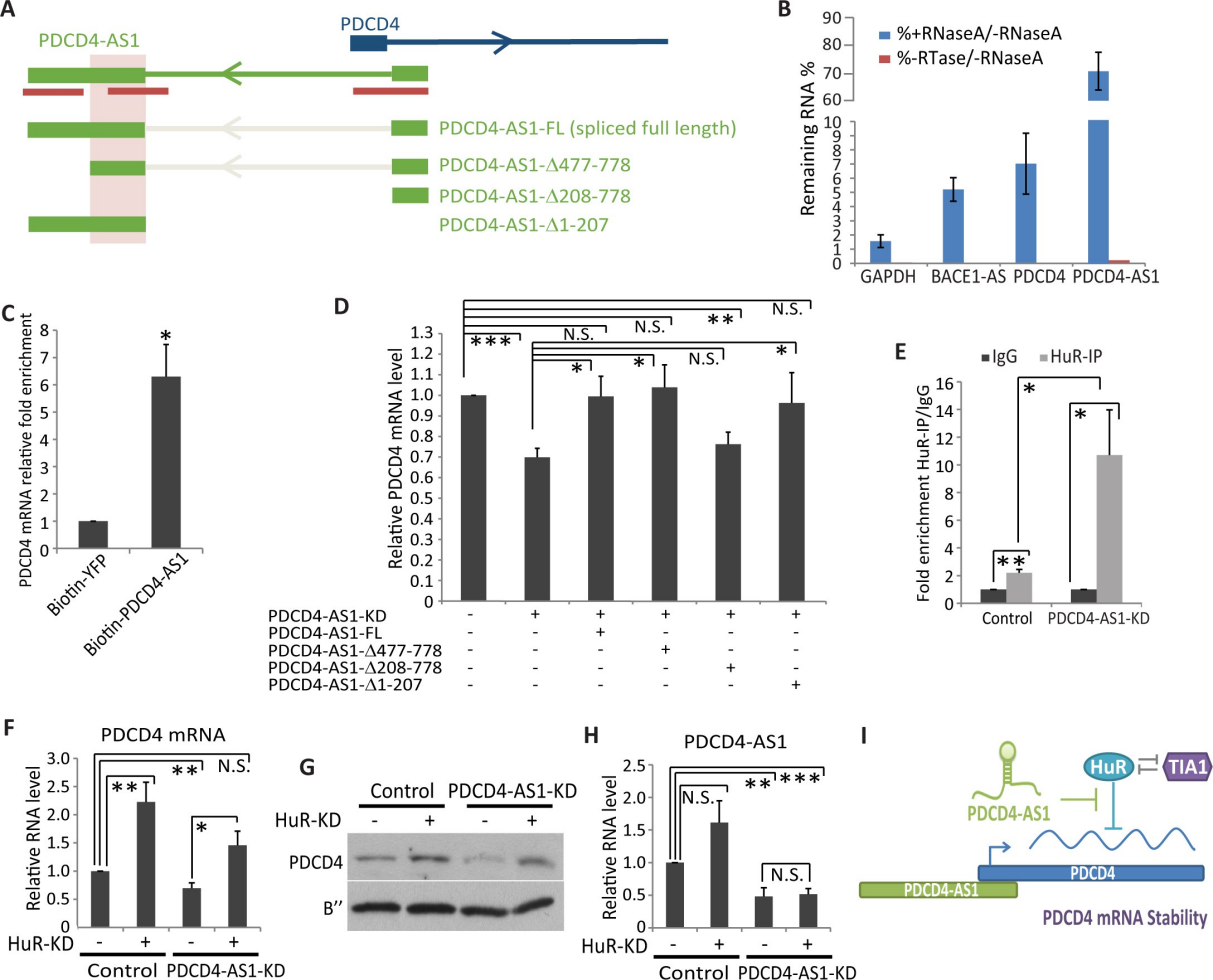
*PDCD4-AS1* forms RNA duplex with *PDCD4* mRNA and regulates the association of RNA decay factors to *PDCD4* mRNA. A) Schematic representation of *PDCD4-AS1/PDCD4* gene locus, and *PDCD4-AS1* full-length and mutants that are used for rescue assay. Red bars show regions of *PDCD4-AS1* with potential complementarity to *PDCD4* mRNA. Shaded region represents the minimum region within *PDCD4-AS1* that is required for stabilizing the level of *PDCD4* mRNA. B) RT-qPCR analyses followed by RNase protection assay. GAPDH is used as negative control where as *BACE-AS1* is used as positive control. C) Affinity RNA pulldown assay followed by RT-qPCR to quantify the interaction between *PDCD*4 and biotin-*PDCD4-AS1*. D) RT-qPCR to quantify the relative levels of *PDCD4* mRNA in *PDCD4-AS1*-depleted M1 cells overexpressing vector alone or other *PDCD4-AS1* constructs. E) RT-qPCR to quantify the levels of *PDCD4* mRNA post HuR-RIP in control and *PDCD4-AS1*-depleted M1 cells. F) RT-qPCR to detect *PDCD4* mRNA level in HuR-depleted control and *PDCD4-AS1*-depleted cells. G) PDCD4 protein level in HuR-depleted control and *PDCD4-AS1*-depleted cells. H) RT-qPCR to detect *PDCD4-AS1* RNA level in HuR-depleted control and *PDCD4-AS1*-depleted cells. I) Proposed model showing the mode of action of *PDCD4-AS1* in promoting the stability of *PDCD4* mRNA by attenuating the association of HuR to the 3UTR of *PDCD4* mRNA. Error bars in (B, C, D, E, F & H) represent mean ± SEM of N≥3 independent experiments (biological replicates). *P<0.05, ** P< 0.01 and ***P<0.001 using Student’s t test. N.S. represents not significant change.

NATs regulate the stability of their sense RNAs by forming RNA duplex [39,40]. Among the several NATs that are involved in conferring mRNA stability, only a few have been shown to form RNA:RNA duplex with their sense RNAs [41,42]. In the case of *PDCD4-AS1/PDCD4* pair, the 5’end of both the transcripts, including exon 1 and part of intron 1, showed complete complementarity (Fig 5A; relative position within *PDCD4-AS1* is highlighted in red lines). In addition, two other repetitive sequence elements located within exon 2 of *PDCD4-AS1* show significant complementarity with sequences within the 3’UTR of *PDCD4* mRNA. A 258 nt long sequence (position 523–778 in exon 2) in *PDCD4-AS1* shows 75% complementarity to a sequence within the 3’UTR *PDCD4* mRNA (position 3164–3417). Besides this one, another shorter repeat of 103 nts long (position 204–306 of exon 2) in *PDCD4-AS1* also shows 82% complementarity with the *PDCD4* mRNA 3’UTR (position 3134–3236) (S4D Fig), indicating that multiple elements within *PDCD4-AS1* and *PDCD4* mRNA could complement to form RNA duplexes. To determine whether *PDCD4-AS1* and *PDCD4* RNA form RNA-duplex under *in vivo* conditions, we initially performed double-strand RNase protection assays as reported earlier [43,44]. RNaseA specifically cleaves the single-stranded RNAs but have no activity on double-stranded/duplex RNAs. RNase protection assays revealed regions within *PDCD4-AS1* and *PDCD4* mRNA that were protected from RNaseA treatment, implying the presence of RNA duplex under *in vivo* conditions (Fig 5B). We used *BACE1/BACE1-AS* pairs as a positive control [43] (Fig 5B). Next, we performed RNA pulldowns followed by RT-qPCR to test physical association between *PDCD4-AS1* and *PDCD4* RNAs [42]. Towards this, we incubated biotin-labeled *PDCD4-AS1* with cell extracts and performed RNA pulldowns using streptavidin-coated beads, followed by RT-qPCR assays. We observed significant interaction between *PDCD4-AS1* and endogenous *PDCD4* RNA in the pulldown experiment (Fig 5C).

Next, we determined to identify sequence elements within *PDCD4-AS1* that play crucial roles in promoting *PDCD4* mRNA stability. To this end, we generated full length and three mutant *PDCD4-AS1* constructs (*PDCD4-AS1*-FL, *PDCD4-AS1*Δ208–778, *PDCD4-AS1*Δ477–778, *PDCD4-AS1*Δ1–207), each of the mutants lacks specific sequence elements that contain *PDCD4* complementary sequences (Fig 5A). We expressed these constructs in control and endogenous *PDCD4-AS1*-depleted M1 cells and determined the effect on endogenous *PDCD4* mRNA levels. RT-qPCR assays in nuclear and cytoplasmic fractionated cell extracts revealed that the transiently expressed full-length and mutant RNAs were localized in both the nucleus and cytoplasm (S4E Fig & S4F Fig). Interestingly, endogenous *PDCD4-AS1*-depleted M1 cells expressing *PDCD4-AS1-FL*, *PDCD4-AS1*Δ1–207 and *PDCD4-AS1*Δ477–778 RNA rescued *PDCD4* mRNA levels (Fig 5D). However, *PDCD4-AS1*Δ208–778 construct, which lacks the second exon of *PDCD4-AS1*, expressing cells failed to rescue the level of *PDCD4* mRNA. Furthermore, RNA stability assays revealed that both *PDCD4-AS1*Δ1–207 and *PDCD4-AS1*Δ477–778 and not *PDCD4-AS1*Δ208–778 rescued the overall stability of *PDCD4* mRNA (S4G Fig). Based on this, we conclude that sequence elements within the exon 2 of *PDCD4-AS1*, which display complementarity to the 3’UTR of *PDCD4* mRNA play crucial roles in stabilizing *PDCD4* mRNA.

Association of RNA-binding proteins (RBPs) to 3’UTRs is known to influence the cellular levels of *PDCD4* mRNA. It was reported recently that RBPs such as HuR (human antigen R) and TIA1 (T-Cell intracellular antigen-1) recognize overlapping sequence within *PDCD4* mRNA 3’UTR, and positively regulate *PDCD4* mRNA levels [45]. Hence, we sought to determine if *PDCD4-AS1* regulates the stability of *PDCD4* mRNA by influencing the binding of these RBPs to *PDCD4* mRNA 3’UTR. ENCODE eCLIP data set identified several potential binding sites of HuR and TIA1 on *PDCD4* RNA [46]. We performed RNA-immunoprecipitation (RIP) under crosslinking conditions using HuR or TIA1 antibody followed by RT-qPCR to determine the interaction between endogenous HuR or TIA1 and *PDCD4* mRNA in control and *PDCD4-AS1*-depleted cells. RIP assays in control cells revealed that both HuR and TIA1 interacted with *PDCD4* mRNA (Fig 5E & S4H Fig). *PDCD4-AS1*-depleted cells showed reduced interaction between TIA1 and *PDCD4* mRNA (S4H Fig). On the contrary, *PDCD4-AS1*-depleted cells showed significantly enhanced interaction between HuR and *PDCD4* mRNA (Fig 5E). Altered interaction of TIA1 or HuR with *PDCD4* mRNA in *PDCD4-AS1*-depleted cells was not due to overall changes in the total cellular levels of RBPs (S4I Fig). Next, we examined if the depletion of HuR and TIA1 would affect the PDCD4 mRNA levels in mammary epithelial cells. Contrary to the earlier report, [45], TIA1-depleted mammary cells did not reduce the levels of *PDCD4* mRNA (S4J Fig & S4K Fig). On the other hand, HuR depletion significantly increased *PDCD4* mRNA and protein levels in control cells, indicating that in mammary epithelial cells HuR negatively regulates the levels of *PDCD4* mRNA (Fig 5F and 5G & S4L Fig). Finally, depletion of HuR in *PDCD4-AS1-*depleted M1 cells rescued the levels of *PDCD4* mRNA and protein (Fig 5F–5H). On the other hand, HuR depletion did not significantly alter the levels of *PDCD4-AS1* RNA, indicating that HuR functions downstream of *PDCD4-AS1* in the *PDCD4-AS1*: *PDCD4*: HuR axis (Fig 5H). Thus, we conclude that *PDCD4-AS1* promotes *PDCD4* mRNA stability by negatively regulating HuR binding to *PDCD4* mRNA. It is likely that the reduced binding of TIA1 to *PDCD4* mRNA in *PDCD4-AS1*-depleted cells is a consequence of enhanced interaction of HuR to the same sequence elements, which also interact with TIA1.

## Discussion

In the present study, we have attempted to understand the involvement of lncRNAs that are differentially expressed in TNBC cell lines, in cancer cell properties. We focused our efforts on NATs and in particular, the roles played by *PDCD4-AS1* in regulating the expression of its sense partner, *PDCD4*. We selected *PDCD4-AS1/PDCD4* pair for mechanistic studies due to the following reasons. First, *PDCD4* is a tumor suppressor gene, and shows reduced expression in several types of cancer, including BC [20,47,48,49,50,51,52,53,54,55,56,57]. Second, both *PDCD4-AS1* and *PDCD4* show concordant expression in BC cell lines and in TNBC patient samples. Finally, clinical survival data in BC patients revealed that similar to *PDCD4* gene, lower expression of *PDCD4-AS1* reduced overall patient survival, implying a tumor suppressor role for *PDCD4-AS1*. PDCD4 is a homolog of eukaryotic translation initiation factor 4G (EIF4G), and by forming a complex with EIF4A1, PDCD4 reduces the interaction between EIF4A1 and EIF4G, thereby inhibiting EIF4A1’s helicase activity. PDCD4 negatively regulates the translation of several oncogenes such as *Cyclins*, *B-Myb* and *c-Myb* [58,59]. Because of its critical role in several vital biological processes, its cellular level under normal physiological conditions is tightly regulated via several transcriptional and post-transcriptional regulatory mechanisms [60,61,62,63,64,65,66]. Our studies, demonstrating the role of *PDCD4-AS1* in enhancing the cellular levels of *PDCD4* adds another layer of complexity in *PDCD4* regulation during BC progression.

NATs are widely present in the human genome, and on an average ~38% of genomic loci in cancer cells express sense: anti-sense pairs [35]. However, NATs are expressed in much lower levels compared to sense transcripts, are mostly enriched in the nucleus, and several of them are shown to influence the expression of their sense partners via *cis-*mediated gene regulation [35]. Similar to earlier observations, we observed aberrant expression of a significant percentage of NATs during BC progression [31,34,35]. Moreover, we observed that several of the NATs expressed from cancer-associated gene loci showed concordant expression with the oncogenic or tumor suppressor sense partner genes and also displayed survival significance in patients, implying their potential involvement in contributing to the molecular pathology of BC progression and or metastasis.

We observed that *PDCD4-AS1* promotes the stability of *PDCD4* mRNA in TNBC cells. *PDCD4-AS1* depletion did not alter *PDCD4* transcription significantly while it compromised the stability of *PDCD4* mRNA. Further, we observed that *PDCD4-AS1* forms RNA duplex with *PDCD4* mRNA, and exon 2 of *PDCD4-AS1* contains sequence elements that promote *PDCD4* mRNA stability. *PDCD4-AS1* could utilize multiple mechanisms to enhance RNA stability. It is possible that by forming RNA duplex, *PDCD4-AS1* could prevent RNase-mediated degradation of *PDCD4* mRNA, as observed in the case of FGFR3-AS1 [67]. Additionally, such RNA duplexes could prevent the binding of miRNAs to the 3’UTR of *PDCD4* mRNA, thereby stabilizing the transcript, as observed in the case of *BACE-AS1/BACE1* pair [33,43]. However, it is quite unlikely that *PDCD4-AS1* promotes *PDCD4* mRNA stability via regulating miRNA binding. Unlike *BACE-AS1*, *PDCD4-AS1* is predominantly localized in the nucleus, and stabilizes nuclear pool of *PDCD4* RNA. A recent study also reported the role of NAT in regulating the expression of its sense partner by modulating chromatin organization [68]. *VIM-AS1* transcribed from *Vimentin (VIM)* gene locus positively regulates *VIM* expression by forming RNA:DNA R-loop structure [68]. Disruption of *VIM-AS1*-mediated R-loop structure compromised *VIM* expression by inducing local chromatin compaction as well as reduced association of transcription factors to *VIM* promoter. In the case of *PDCD4-AS1*, its depletion did not significantly change *PDCD4* transcription, indicating that *PDCD4-AS1* might not act *via* such a mechanism.

Alternatively, *PDCD4-AS1* by forming RNA duplex with *PDCD4* RNA could influence the binding of RNA-binding proteins (RBPs) to *PDCD4* mRNA. We observed that *PDCD4-AS1* negatively regulates the association of HuR with *PDCD4* mRNA. HuR-depletion studies in M1 cells further identified HuR as a destabilizer of *PDCD4* mRNA. HuR/ELAVL1 is a U-/AU-rich element interacting RBP that is known to regulate mRNA stability. (For review on HuR in breast cancer cells please see [69]. Several recent studies have described the role of HuR in destabilizing RNAs [70,71,72,73,74]. For example, HuR utilizes AUF1, Ago2 or let-7 miRNA as co-factors to enhance the decay of *p16(INK4)* and *MYC* mRNAs [73,74]. HuR also promotes the early steps of myogenesis by destabilizing *nucleophosmin*/*NPM* mRNA [72]. We recently reported that in mouse cells, double stranded RNA binding protein ADAR1 & 2 negatively regulates HuR-mediated degradation of a significant number of RNAs [70,71]. Earlier studies have observed that NATs by forming RNA duplex with regions of mRNA containing AU-rich sequences, influences that association of AU-rich interacting RNA decay factors, thereby controlling mRNA stability [75,76]. For example, an antisense RNA from *HIF1α* locus destabilizes one of the isoforms of HIF1α by binding to it and exposing the AU-rich sequence element within the HIF1α 3’UTR [76]. On the other hand, a NAT transcribed from the *Bcl2/IgH* hybrid gene stabilizes the mRNA by masking the AU-rich sequence element [75]. In the present study, we observed that HuR destabilizes *PDCD4* mRNA. The molecular mechanism underlying *PDCD4-AS1*-mediated inhibition of HuR/*PDCD4* RNA interactions remained to be determined. It is known that a significant proportion of HuR is localized in the nucleus, and we have previously shown that nuclear pool of HuR destabilizes RNA [70,71]. Based on this, we hypothesize that the formation of RNA duplex between *PDCD4-AS1* and *PDCD4* RNA in the nucleus occludes the binding of HuR to the *PDCD4* RNA, thereby stabilizing *PDCD4* RNA (Fig 5I).

At present, it is not clear how NATs, which in general are present in lower copy numbers (~10–100 fold) than their sense protein coding transcripts modulate post-transcriptional RNA processing in *cis* [35]. For example, *Wrap53*, a NAT that is expressed at 100-fold lower levels than its sense partner, the tumor suppressor *p53* gene, positively regulates the stability of *p53* mRNA [42]. Similarly, low copy NAT, *iNOS-AS* (expressed in 7 fold lower) transcribed from *iNOS* locus interacts with the 3’UTR of *iNOS* RNA and positively regulates its stability [77,78]. As a matter of fact, the question of how low copy NATs regulate post-transcriptional processing of their sense transcripts remains an “unresolved conundrum” in the antisense-RNA field [79]. At present, there is no convincing molecular explanation of how NATs regulate the stability of high copy sense RNAs. Several studies have posed models to explain potential mode of action [42,80]. It is suggested that transient association of NAT with its sense RNA allows one NAT molecule to interact with multiple sense transcripts in a ‘hit and run’ fashion [42]. Such interactions could initiate local changes in sense RNA structure that favor or inhibit the binding of RBPs [42]. In a “recycling hypothesis” model, short complementary regions within the sense RNA:NAT pair promote intermolecular RNA:RNA interactions [80]. These interactions are transient and unstable due to the low melting temperature of the small duplex, and trigger conformational changes in the sense RNA, allowing either enhanced accessibility of a stabilizing RNA-binding protein or decreased affinity of an RNA decay factor to RNA, thereby modulating RNA stability. Once an RNP complex is formed, and the sense RNA is stabilized, the NAT is released from the complex and is recycled to stabilize another RNA molecule [80]. We observed that *PDCD4-AS1* is expressed ~18 fold lower than *PDCD4* mRNA in total cell extracts. However *PDCD4-AS1*/*PDCD4* ratio in the nucleus, especially at their site of transcription will be much higher due to the fact that a major fraction of *PDCD4-AS1* is enriched in the nucleus, where as most of the *PDCD4* mRNA is exported to the cytoplasm. Based on these data, we hypothesize that transient interaction between *PDCD4-AS1* and *PDCD4* RNA in the nucleus, preferentially at the site of transcription, trigger conformational changes in *PDCD4* RNA, resulting in differential binding of RBPs, such as HuR (decay factor) and AUF1 (stabilizing factor) to *PDCD4* RNA. In this scenario, a single *PDCD4-AS1* RNA could interact with several *PDCD4* RNAs during its lifetime. In general, our studies have underscored the importance of a NAT in BC progression *via* its role in regulating the expression of a tumor suppressor sense partner. Future studies will unravel mechanistic roles of hundreds of other BC-deregulated lncRNAs in breast cancer biology.

## Material and methods

### Ethics statement

All of the patient RNA-seq data was obtained from the publicly available database, TCGA (https://cancergenome.nih.gov/), and no additional ethics approval was needed.

### 3D acini culture of M1-M4 cells

Acinar culture of M1-M4 cells was performed similar to three-dimensional culture of MCF10A cells described elsewhere [30]. Briefly, growth-factor reduced Matrigel was used to coat multi-well plates. A single-cell suspension of each of the cell lines M1-M4 was prepared. M2-M2 cells were suspended in an assay medium containing growth medium (DMEM/F12 containing 2% Horse serum, 1 mg/ml hydrocortisone, 1 mg/ml cholera toxin, 10 mg/ml insulin, 10 ng/ml EGF, and 1% penicillin/streptomycin as well as 2.5% Matrigel dissolved in the medium. M3-M4 cells are prepared in the same way but omitting the EGF in the medium. The cells were seeded at a concentration of 8000 cells/mL. Media was changed every fourth day. Cells were cultured for 8 days prior to harvesting.

### 2D cell culture

M1 and M2 cells were cultured in DMEM/F12 medium containing 5% horse serum supplemented with 100 U/mL penicillin, 100μg/mL streptomycin, 20ng/mL EGF (epidermal growth factor), 0.5 μg/mL Hydrocortisone, 100ng/mL Cholera toxin, 10 μg/mL insulin and 5% horse serum. M3 and M4 cells were cultured DMEM/F12 medium containing 5% horse serum supplemented with 100 U/mL penicillin, 100μg/mL streptomycin.

### RNA-seq of M1-M4 cells and bioinformatics analysis

8 (biological replicates of M1-M4) poly A+ RNA samples were pooled and sequenced in two lanes on HiSeq using Illumina TruSeq mRNA Prep Kit RS-122-2101 and paired-end sequencing. The samples have 163 to 256 million pass filter reads with a base call quality of above 94% of bases with Q30 and above. Reads of the samples were trimmed for adapters and low-quality bases using Trimmomatic software before alignment with the reference genome (Human—hg19) and the annotated transcripts using STAR. The average mapping rate of all samples is 96%. Unique alignment is above 87%. There are 3.74 to 4.07% unmapped reads. The mapping statistics are calculated using Picard software. The samples have 0.59% ribosomal bases. Percent coding bases are between 67–72%. Percent UTR bases are 23–26%, and mRNA bases are between 94–96% for all the samples. Library complexity is measured in terms of unique fragments in the mapped reads using Picard’s MarkDuplicate utility. The samples have 31–52% non-duplicate reads. In addition, the gene expression quantification in raw count format was performed for all samples using STAR/RSEM tools by the annotation of Gencode v19 and normalized by size factor implemented in DESeq2 package. We calculated the fold change gene expression based on FPKM data. We identified deregulated genes with >2 fold cut off and then made the overlap list between two biological repeats. RNA seq data is deposited to GEO (GEO accession number GSE120796).

### RNA extraction, RT PCR and quantitative PCR

Trizol reagent (Invitrogen) was used to extract total RNA according to manufacturer’s protocol. The concentration was measures using Nanodrop instrument (ThermoFisher SCIENTIFIC). RNA was treated with RNase-free DNase I (Sigma, USA) and cDNA was synthesized from RNA using High capacity reverse transcription kit (Applied Biosystem). Quantitative PCR was carried out by StepOnePlus system (Applied Biosystem). For gene specific primers please see S8 Table.

### Knockdown/overexpression experiments

*PDCD4* depletion was achieved by transfection with siRNA against GL3 (control) or siRNAs against *PDCD4* (40–50 nM con, IDT) for one round using Lipofectamine RNAiMax reagent (Invitrogen). *TIA1* depletion was performed using siRNA purchased from IDT. *HuR* depletion was carried out using siRNA as used in [81]. *PDCD4-AS1* knockdown was performed by shRNA lentivirus-mediated transduction. *PDCD4-AS1* depletion was achieved using gapmer ASOs at 200 nM final concentration (Ionis Pharmaceuticals Inc.). For overexpression, full-length *PDCD4* was purchased as pGEX6p1-*hPdcd4* from Addgene [82] and cloned into pCGT vector. Full length *PDCD4-AS1* and mutants were purchased as gblocks from IDT technology, cloned and expressed in pCGT vector, and empty vector was used as control.

### Cell migration assay

We used transwell migration chambers (Corning, Cat# 354578) and to perform migration assays as previously explained [17]. Briefly, cells were starved in a serum-free medium, which was then trypsinized, counted and seeded in serum-free medium in transwell chamber (8μM). We placed the cell containing chambers into a well containing serum (24-well plate). Cells were kept in incubator 37 C, 5% CO2 overnight. Migrating cells were stained by Crystal Violet 0.05% and counted the day after.

### Wound healing assay

The wound was created by 200 μl filter tips. After washing with PBS, serum-free medium was added to cells in order to discourage the cell proliferation. Images were taken at Day0, 1, 2 and 3 after wound creation to monitor the wound healing.

### Nascent RNA capture assay

Click-iT Nascent RNA capture kit (Invitrogen, Cat # C10365) was used to isolate nascent RNA following the product’s protocol. Then quantitative RT-qPCR was performed using gene-specific primers.

### RNA stability assay

Cells were treated with Flavopiridol (1M) and were collected at different time points post treatment. RNA extraction and RT-qPCR was carried out as explained above.

### RNA immunoprecipitation (RIP)

RIP was conducted as described before [71,83]. Briefly, RNA-Protein interactions were reversibly crosslinked by formaldehyde in cells. Cells were lysed and lysate was immunoprecipitated using Anti-HUR (HuR (3A2): sc-5261, Santa Cruz Biotechnology) and Anti-TIA1 antibody (TIA-1 (G-3): sc-166247, Santa Cruz Biotechnology). After RIP, we reversed cross-link and RNA extraction using Trizol LS (Invitrogen). DNase I treatment, reverse transcription and qPCR was performed as described above.

### Nuclear and cytoplasmic fractionation

As explained in [71], we washed cells with PBS and lysed in RSB buffer (10 mM Tris-HCl pH7.4, 100 mM NaCl, 2.5 mM MgCl2, RNase Inhibitor) supplemented with Digitonin (8 g/ml) (D141-100MG, Sigma-Aldrich, USA) for 10 min on ice. Lysate was centrifuged at 2000 rpm, 4°C, 10 min. Supernatant was collected as cytoplasmic fraction and RNA was extracted from with Trizol LS (Invitrogen). The pellet included the nuclear fraction. We washed the nuclear pellet with RSB-Digitonin solution one more time and then RNA was extracted using Trizol reagent (Invitrogen).

### Poly(A) fractionation

Poly(A) fractionation was performed as previously described [44]. In brief, NucleoTrap mRNA kit (Clontech) was used to fractionate Poly(A) plus and Poly(A) minus fractions following by extraction, RT and qPCR.

### RNase protection assay

The experiment was performed as described previously [44]. Cells were washed with PBS and lysed in lysis buffer (10 mM Tris pH 7.4, 3 mM CaCl2, 2 mM MgCl2, and 0.7% NP-40). Cell lysate was passed through needle (27.5 gauge) five times and then incubated on ice for 10 minutes. The final solution was adjusted to DNase I (Sigma) 12.5 units/ml and 125 mM NaCl. The lysate was divided to two fractions. To one fraction RNase A (QIAgen) and the other fraction RNAse Inhibitor was added to final concentrations of 200 ng/ml and 250 units/ml; respectively. Then, solutions were incubated at 37°C for 40 minutes. RNA was extracted using Trizol LS (Invitrogen).

### Biotin RNA pulldown assays

*PDCD4-AS1* and YFP (-ve control) full-length cDNA cloned in pGEM-Teasy plasmids were *in vitro* transcribed to generate biotinylated RNA (Biotin Labeling Mix; Roche). M1 whole cell extract was incubated with biotin-labeled transcripts followed by streptavidin-mediated RNA pull down. Then RNA extraction, RT-PCR and qPCR were performed to analyze potential RNA: RNA interactions.

## Supporting information

S1 Fig *PDCD4-AS1* lncRNA and *PDCD4* show reduced expression during BC progression. A) RNAseq signals, corresponding to *PDCD4-AS1* in UCSC browser tracks in M1, M2, M3 and M4 cells. Signal intensity is adjusted (0–20). B) RNAseq signals, corresponding to *PDCD4* in UCSC browser tracks in M1, M2, M3 and M4 cells. Signal intensity is adjusted (0–1000). C) *PDCD4* RNA level in various subclasses of breast cancer samples. D) *PDCD4* RNA level in various stages of breast cancer patient samples. E) Kaplan–Meier analysis to depict the survival rate in TCGA breast cancer patients with high, medium and low levels of normalized expression of *PDCD4*. In C-E, we retrieved *PDCD4* expression data from TCGA-BRCA- gene expression- Illumina-HiSeq from UCSC Xena portal. The TCGA data statistical analysis was done using UCSC Xena portal. For plotting, raw data was downloaded from Xena and plotted using R. F) RNAseq signal in M1, corresponding to *PDCD4-AS1* annotation in UCSC browser. G) Coding probability of several RNA, including PDCD4-AS1, calculated by coding potential Assessing Tool (CPAT).
(TIF)

S2 Fig *PDCD4* and *PDCD4-AS1* show induction during cellular quiescence. A) Flow cytometry analyses of Asynchronous and quiescent M1 cells. B) Percentage of cells at different cell cycle stage in asynchronous and quiescent M1 cells, observed by flow cytometry analyses. C) *PDCD4 and PDCD4-AS1* relative RNA levels in asynchronous and quiescent M1 cells. D) PDCD4 protein levels in biologically triplicate asynchronous and quiescent M1 cells. Error bars in (B) represent mean ± SEM of three independent experiments (biological replicates).
(TIF)

S3 Fig A) Schematic representation of *PDCD4-AS1/PDCD4* gene locus, showing the position of three shRNAs (sh1-3) utilized to stably deplete *PDCD4-AS1*. B) RT-qPCR reveals significant depletion of *PDCD4-AS1* RNA in cells stably transfected with *PDCD4-AS1* shRNAs. C) RT-qPCR reveals significant depletion of *PDCD4-AS1* RNA in both nuclear and cytoplasmic fractions in M1 cells. D) RT-qPCR reveals significant depletion of *PDCD4-AS1* and *PDCD4 RNAs* in cells transfected with modified DNA antisense oligonucleotides (gapmers) against *PDCD4-AS1*. E) Cell cycle flow cytometry in control and *PDCD4-AS1* depleted M1 cells. F) RT-qPCR reveals significant depletion of *PDCD4* RNA upon PDCD4-AS1 KD in both nuclear and cytoplasmic fractions in M1 cells. Error bars in B represent mean ± SEM of N≥3 independent experiments (biological replicates). *P<0.05, ** P< 0.01 and ***P<0.001 using Student’s t test.
(TIF)

S4 Fig *PDCD4-AS1* regulates the stability of *PDCD4* mRNA by influencing the association of RNA decay factors. A) PDCD4 immunoblot in cells transfected with vector or PDCD4 cDNA containing plasmid and transwell migration assay in control and *PDCD4*-overexpressing M1 cells. B) RT-qPCR to quantify the relative levels of *SHOC2, BBIP1, and RBM20* mRNA in control and *PDCD4-AS1* depleted M1 cells. C) RT-qPCR to quantify the relative levels of *PDCD4, SHOC2, BBIP1, RBM20* mRNA levels in control and *PDCD4* depleted M1 cells. D) *PDCD4* mRNA dot plot alignment with non-spliced *PDCD4-AS1* showing three potential complementarity regions. E) RT-qPCR to quantify the relative levels of *PDCD4-AS1* full-length and mutant RNA in endogenous *PDCD4-AS1*-depleted M1 cells overexpressing *PDCD4-AS1* constructs. F) RT-qPCR analyses in nuclear and cytoplasmic fractionated RNA from M1 cells overexpressing *PDCD4-AS1* constructs. G) RT-qPCR to quantify *PDCD4* mRNA stability assay using RNA from control and *PDCD4-AS1*-depleted M1 cells overexpressing *PDCD4-AS1* constructs treated with Flavopiridol (1M) for indicated time points. H) RT-qPCR to quantify the levels of *PDCD4* mRNA in IgG and TIA1 RIP in control and *PDCD4-AS1* depleted M1 cells. I) Immunoblot to detect TIA1 protein in control and *PDCD4-AS1* depleted M1 cells. J) TIA1 protein and K) *PDCD4* mRNA level in control and *TIA1*-depleted M1 cells. L) Immunoblot to detect HuR protein in control and HuR-depleted M1 cells. B”-U2snRNP is used as a loading control (A, I, J & L). Error bars in (B, C & G) represent mean ± SEM of three independent experiments (biological replicates).
(TIF)

S1 Table Gene expression in M1, M2, M3 and M4 cells. (XLSX)

S2 Table Genes deregulated in M4 compared to M1. (XLSX)

S3 Table List of NAT-PC (protein coding) gene pairs. (XLSX)

S4 Table Deregulated NAT-PC (protein coding) gene pairs in M4 compared to M1. (XLSX)

S5 Table Deregulated NAT-cancer-associated PC (protein coding) gene pairs in M4 compared to M1. (XLSX)

S6 Table List of cancer-associated genes. (XLSX)

S7 Table Deregulated NAT-cancer-associated PC (protein coding) gene pairs in M4 compared to M1 cells where NAT expression is significantly correlated with survival rate in TCGA breast invasive carcinoma patients. (XLSX)

S8 Table List of oligonucleotides, primers and siRNAs. (XLSX)
