# Supplementary figures and images for: A natural antisense lncRNA controls breast cancer progression by promoting tumor suppressor gene mRNA stability
Source: PLoS Genet. 2018 Nov 29;14(11):e1007802. doi: 10.1371/journal.pgen.1007802 (PMC6289468; doi:10.1371/journal.pgen.1007802)

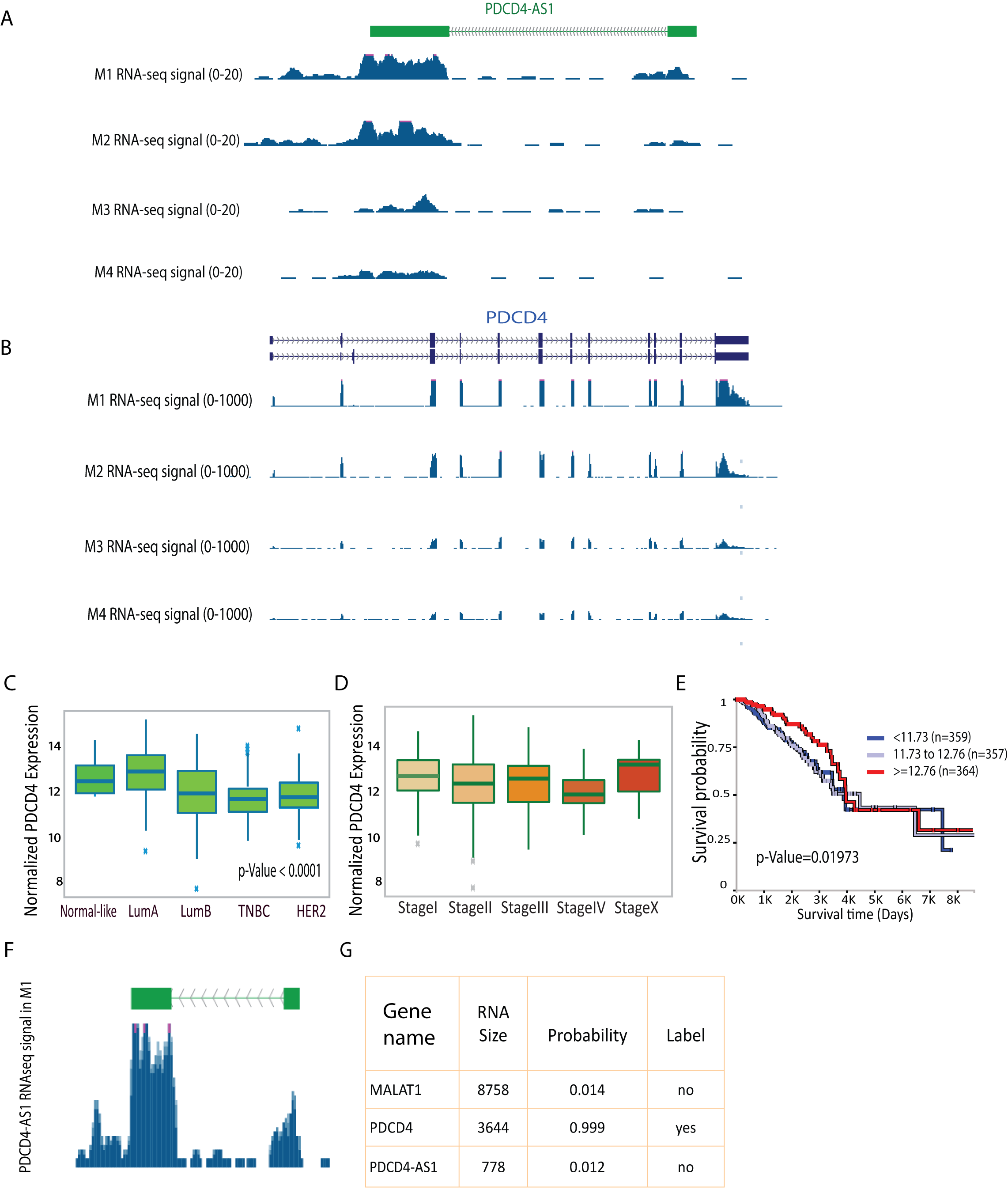

Supplement: S1 Fig — A) RNAseq signals, corresponding to PDCD4-AS1 in UCSC browser tracks in M1, M2, M3 and M4 cells. Signal intensity is adjusted (0–20). B) RNAseq signals, corresponding to PDCD4 in UCSC browser tracks in M1, M2, M3 and M4 cells. Signal intensity is adjusted (0–1000). C) PDCD4 RNA level in various subclasses of breast cancer samples. D) PDCD4 RNA level in various stages of breast cancer patient samples. E) Kaplan–Meier analysis to depict the survival rate in TCGA breast cancer patients with high, medium and low levels of normalized expression of PDCD4. In C-E, we retrieved PDCD4 expression data from TCGA-BRCA- gene expression- Illumina-HiSeq from UCSC Xena portal. The TCGA data statistical analysis was done using UCSC Xena portal. For plotting, raw data was downloaded from Xena and plotted using R. F) RNAseq signal in M1, corresponding to PDCD4-AS1 annotation in UCSC browser. G) Coding probability of several RNA, including PDCD4-AS1, calculated by coding potential Assessing Tool (CPAT). (TIF) [file pgen.1007802.s001.tif]

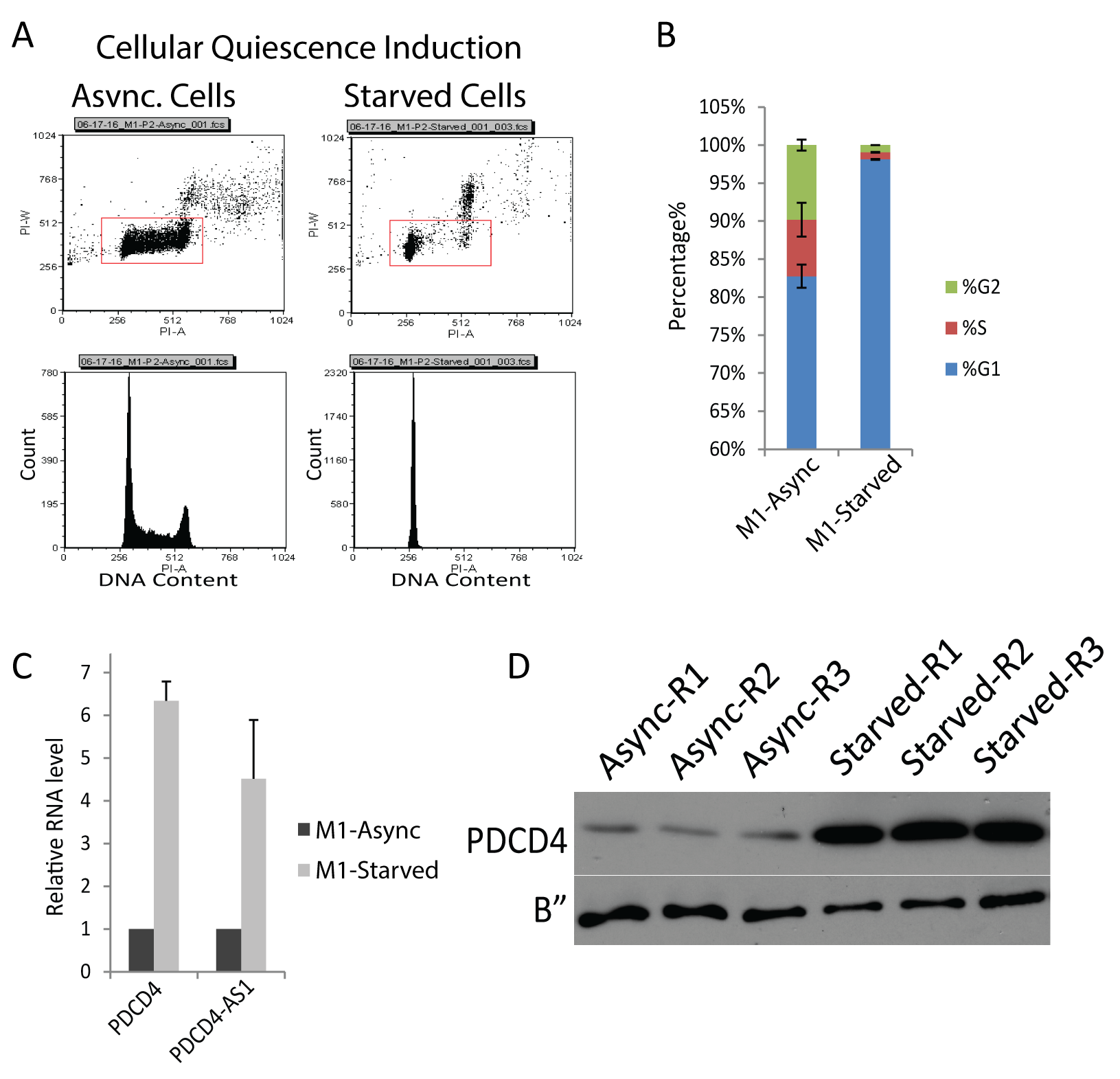

Supplement: S2 Fig — A) Flow cytometry analyses of Asynchronous and quiescent M1 cells. B) Percentage of cells at different cell cycle stage in asynchronous and quiescent M1 cells, observed by flow cytometry analyses. C) PDCD4 and PDCD4-AS1 relative RNA levels in asynchronous and quiescent M1 cells. D) PDCD4 protein levels in biologically triplicate asynchronous and quiescent M1 cells. Error bars in (B) represent mean ± SEM of three independent experiments (biological replicates). (TIF) [file pgen.1007802.s002.tif]

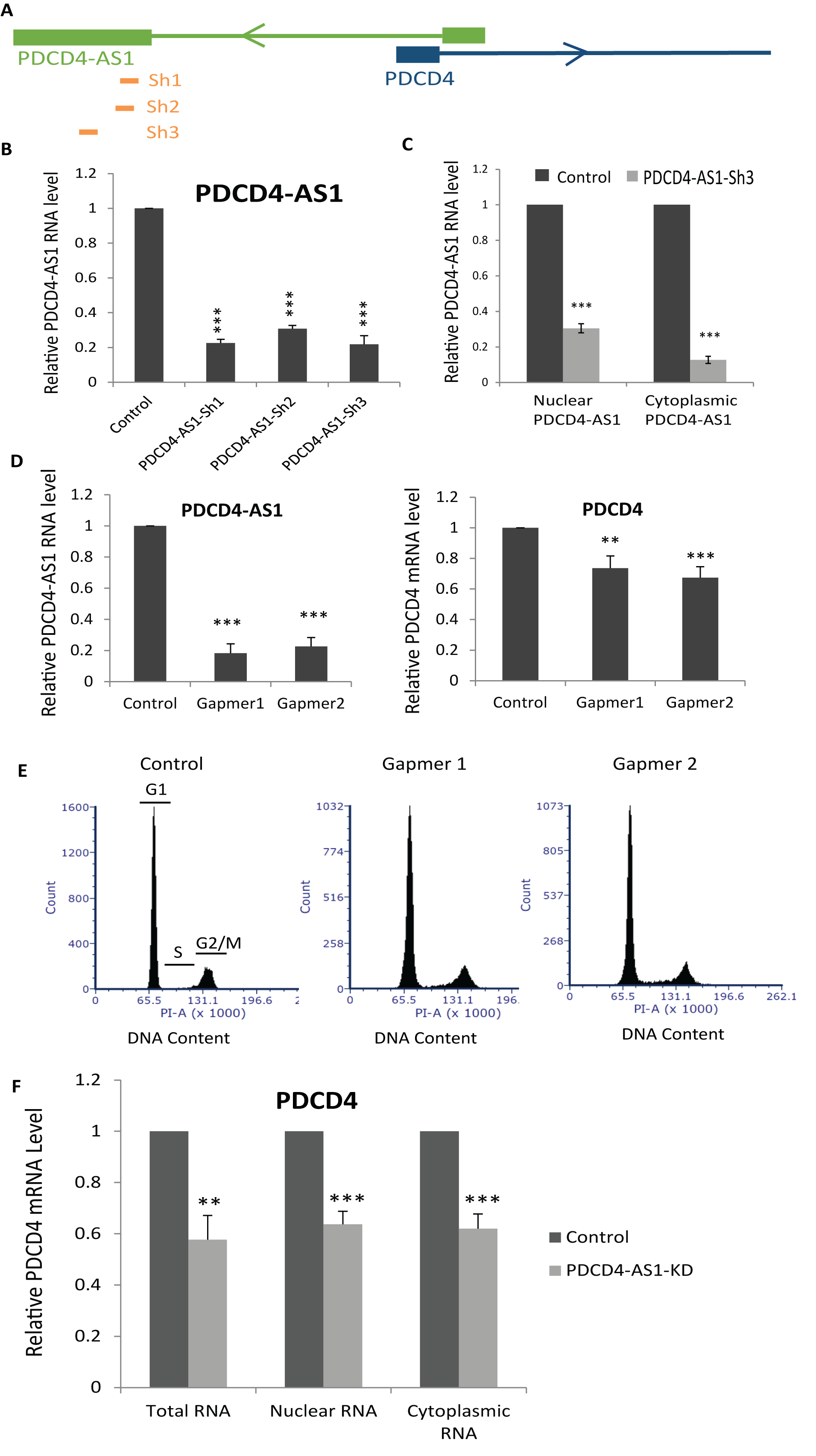

Supplement: S3 Fig — A) Schematic representation of PDCD4-AS1/PDCD4 gene locus, showing the position of three shRNAs (sh1-3) utilized to stably deplete PDCD4-AS1. B) RT-qPCR reveals significant depletion of PDCD4-AS1 RNA in cells stably transfected with PDCD4-AS1 shRNAs. C) RT-qPCR reveals significant depletion of PDCD4-AS1 RNA in both nuclear and cytoplasmic fractions in M1 cells. D) RT-qPCR reveals significant depletion of PDCD4-AS1 and PDCD4 RNAs in cells transfected with modified DNA antisense oligonucleotides (gapmers) against PDCD4-AS1. E) Cell cycle flow cytometry in control and PDCD4-AS1 depleted M1 cells. F) RT-qPCR reveals significant depletion of PDCD4 RNA upon PDCD4-AS1 KD in both nuclear and cytoplasmic fractions in M1 cells. Error bars in B represent mean ± SEM of N≥3 independent experiments (biological replicates). *P<0.05, ** P< 0.01 and ***P<0.001 using Student’s t test. (TIF) [file pgen.1007802.s003.tif]

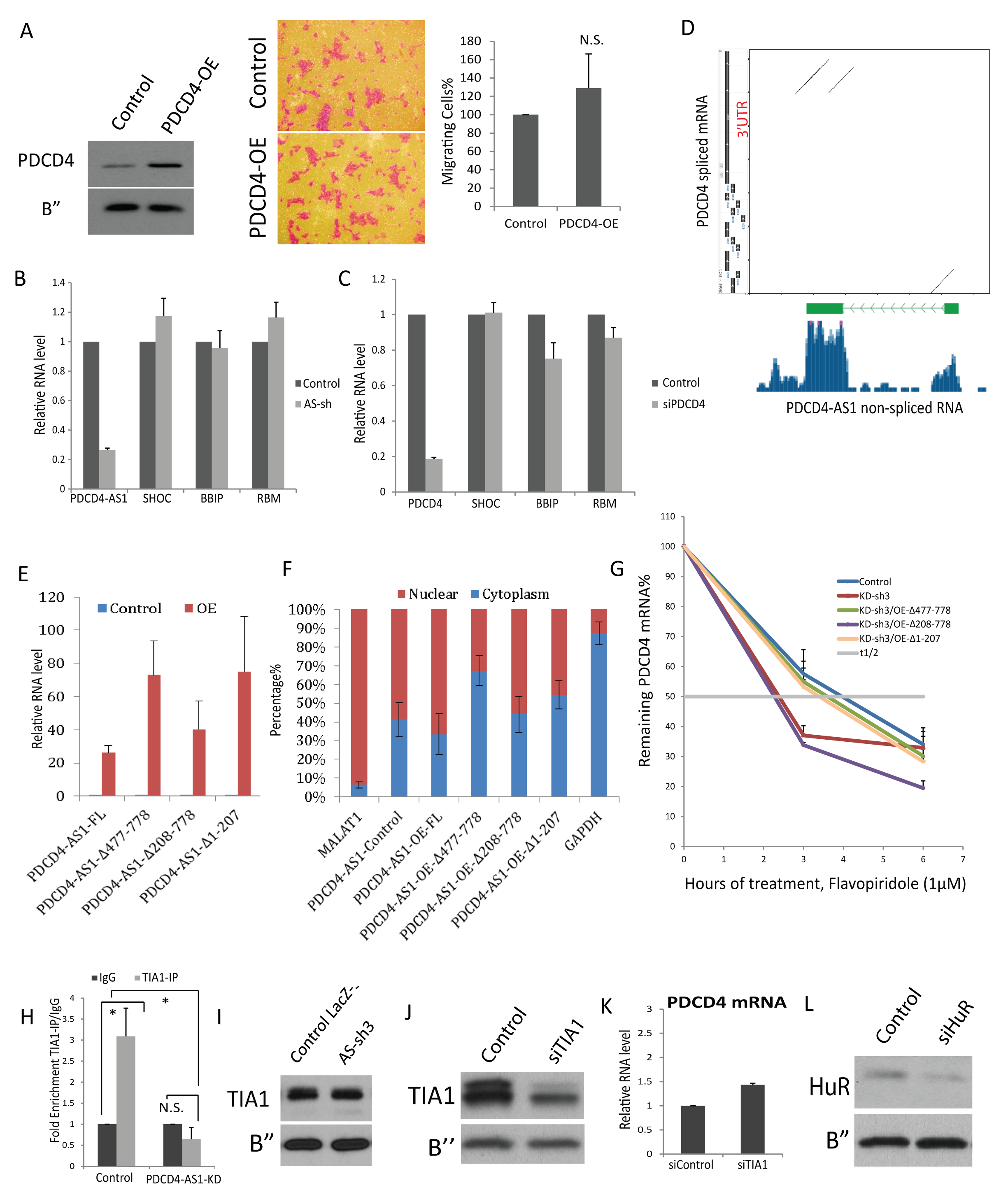

Supplement: S4 Fig — A) PDCD4 immunoblot in cells transfected with vector or PDCD4 cDNA containing plasmid and transwell migration assay in control and PDCD4-overexpressing M1 cells. B) RT-qPCR to quantify the relative levels of SHOC2, BBIP1, and RBM20 mRNA in control and PDCD4-AS1 depleted M1 cells. C) RT-qPCR to quantify the relative levels of PDCD4, SHOC2, BBIP1, RBM20 mRNA levels in control and PDCD4 depleted M1 cells. D) PDCD4 mRNA dot plot alignment with non-spliced PDCD4-AS1 showing three potential complementarity regions. E) RT-qPCR to quantify the relative levels of PDCD4-AS1 full-length and mutant RNA in endogenous PDCD4-AS1-depleted M1 cells overexpressing PDCD4-AS1 constructs. F) RT-qPCR analyses in nuclear and cytoplasmic fractionated RNA from M1 cells overexpressing PDCD4-AS1 constructs. G) RT-qPCR to quantify PDCD4 mRNA stability assay using RNA from control and PDCD4-AS1-depleted M1 cells overexpressing PDCD4-AS1 constructs treated with Flavopiridol (1M) for indicated time points. H) RT-qPCR to quantify the levels of PDCD4 mRNA in IgG and TIA1 RIP in control and PDCD4-AS1 depleted M1 cells. I) Immunoblot to detect TIA1 protein in control and PDCD4-AS1 depleted M1 cells. J) TIA1 protein and K) PDCD4 mRNA level in control and TIA1-depleted M1 cells. L) Immunoblot to detect HuR protein in control and HuR-depleted M1 cells. B”-U2snRNP is used as a loading control (A, I, J & L). Error bars in (B, C & G) represent mean ± SEM of three independent experiments (biological replicates). (TIF) [file pgen.1007802.s004.tif]
